# Supplementary material for: Role of Two Plant Growth-Promoting Bacteria in Remediating Cadmium-Contaminated Soil Combined with Miscanthus floridulus (Lab.)
Source: Plants (Basel). 2021 May 2;10(5):912. doi: 10.3390/plants10050912 (PMC8147505; doi:10.3390/plants10050912)
Supplement: Supplementary file 1 [file plants-10-00912-s001.zip › Table S1.pdf]

Table 1 Contents of Cd in reference materials

Metal	Reference material certified content		Digestion in our laboratory		
	mg/kg	uncertainty	mg/kg	recovery (%)	difference (%)
Cd	6.10	0.70	5.83	95.57%	-4.43%
