# Supplementary figures and images for: Role of Two Plant Growth-Promoting Bacteria in Remediating Cadmium-Contaminated Soil Combined with Miscanthus floridulus (Lab.)
Source: Plants (Basel). 2021 May 2;10(5):912. doi: 10.3390/plants10050912 (PMC8147505; doi:10.3390/plants10050912)

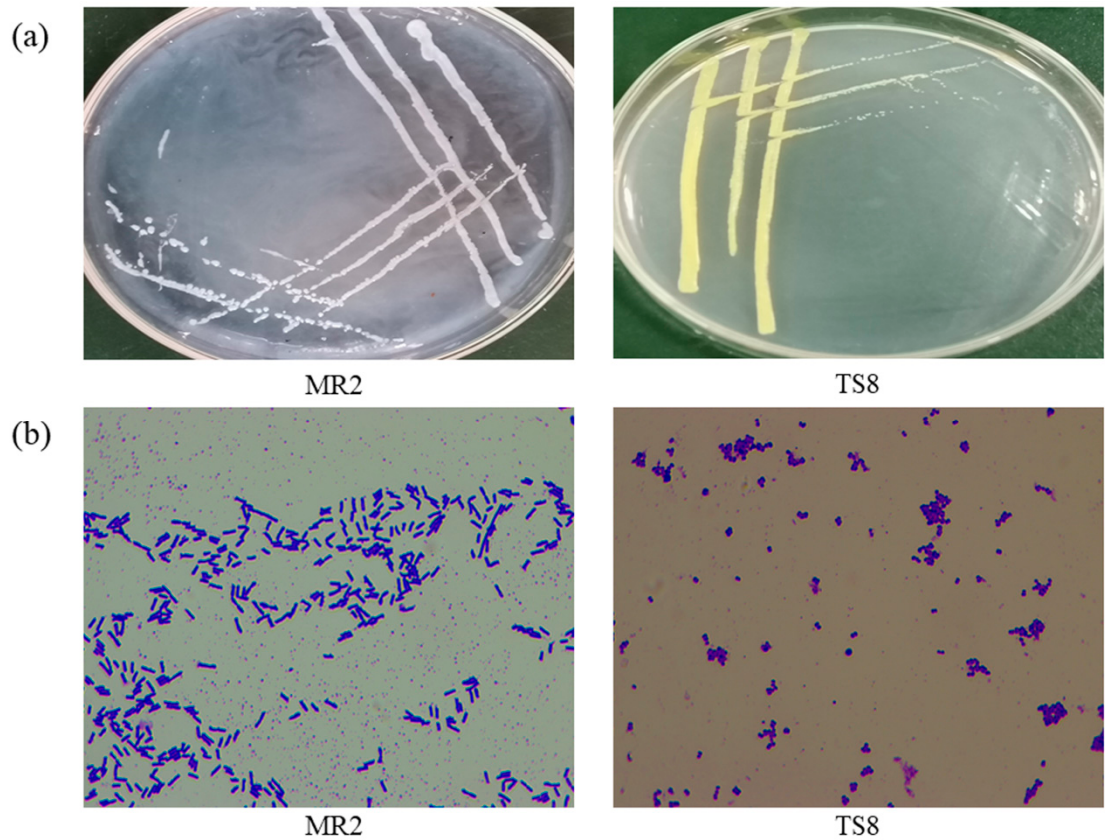

Figure S2. Results of selected PCGPB morphological identifications. (a) plate; (b) simple

Supplement: Supplementary file 1 [file plants-10-00912-s001.zip › Figure S2.pdf]
